# Plasma Elastase Screening in Hematological Disease Reveals Its Potential as a Diagnostic and Prognostic Biomarker in Hematological Malignancies

**DOI:** 10.1111/ijlh.70090

**Published:** 2026-03-12

**Authors:** Pasqualina Scala, Anna Maria Della Corte, Angela Bertolini, Marisa Gorrese, Francesca Picone, Bianca Serio, Danilo De Novellis, Laura Mettivier, Luca Pezzullo, Maria Carmen Martorelli, Denise Morini, Matteo D'Addona, Bianca Cuffa, Carmine Selleri, Valentina Giudice

**Affiliations:** ^1^ Department of Medicine, Surgery, and Dentistry University of Salerno Baronissi Italy; ^2^ Hematology and Transplant Center University Hospital “San Giovanni di Dio e Ruggi d'Aragona” Salerno Italy

**Keywords:** elastase, hematological diseases, NETosis, NETs, neutrophils

## Abstract

**Introduction:**

Neutrophil‐extracellular traps are net‐like material released by triggered neutrophils and composed of decondensed chromatin linked to nuclear proteins. Elastase, one of the fourth most represented neutrophil‐specific serine proteases stored in azurophil granules of naïve neutrophils, exerts various actions, including degradation of extracellular matrix and has proinflammatory functions.

**Methods:**

Plasma was obtained from 111 patients with various hematological malignancies and 42 healthy donors, and plasma elastase levels were measured by ELISA.

**Results:**

Reduced circulating levels of neutrophil elastase were found in patients with myelodysplastic syndromes (MDS) and chronic lymphocytic leukemia, while they were increased in acute myeloid leukemia (AML) and non‐Hodgkin lymphomas (NHL), with statistically significant AUCs (AML, AUC = 0.7821 and *p* = 0.0182; and NHL, AUC = 0.7521 and *p* = 0.0008). Moreover, multiple patients with standard‐risk genetic features tended to have higher plasma elastase levels compared to healthy controls. Patients were then divided into two groups, using a cut‐off of 300 ng/mL of plasma elastase, and clinical outcomes were compared, showing reduced overall survival and progression‐free survival in those subjects with increased plasma elastase levels, as well as shorter time‐to‐treatment.

**Conclusion:**

Our findings indicate that circulating plasma elastase could be useful to distinguish across diseases in the differential diagnosis of hematological malignancies, and could be used as additional prognostic biomarker of disease progression and responsiveness to therapies.

## 1 Introduction

Neutrophils, the most abundant circulating polymorphonuclear cell population in the peripheral blood (PB), are mainly involved in innate immune responses against pathogens, as they are specialized in phagocytosis and secretion of cytotoxic enzymes, reactive oxygen species, chemokines, and cytokines from their cytoplasmic granules [3]. Another mechanism of pathogen elimination is the release of neutrophil extracellular traps (NETs), through a process called NETosis. During this release process, nuclear and cytoplasmic materials are spread into the extracellular matrix by activated neutrophils, to entrap and neutralize pathogens, acting like glue [4]. NETosis material includes web‐like decondensed chromatin with 200 nm diameter pores linked to nuclear proteins, including histones, antimicrobial peptides, and proteins packed into cytosol and granules, including elastase, cathelicidin, cathepsin G, and myeloperoxidase (MPO) [5, 6]. Elastase, one of the four most abundant neutrophil‐specific serine proteases stored in azurophil granules of naïve neutrophils, facilitates degradation of extracellular matrix, plasma proteins, and pro‐inflammatory mediators. During immune responses, elastase has antimicrobial activity by recognizing pathogen‐related molecules and by neutralizing bacteria and fungi [7]. Moreover, elastase is crucial for NETs formation, as it translocates to the nucleus within 60 min from stimulation, where it cleaves and digests nucleosomal histones for chromatin decondensation [8, 9]. However, this process must be finely tuned, as an over‐release boosts uncontrolled inflammatory responses, and excessive NETosis is related to cancer progression, metastasis development, autoimmune disorders, and thrombosis [6, 10, 11].

In hematological malignancies, NETosis is increased in chronic myeloid leukemia (CML) at diagnosis, as well as baseline elastase levels and after neutrophil stimulation compared to healthy subjects [12]. Similarly, NETosis is higher in chronic lymphocytic leukemia (CLL) patients than healthy controls (HC), and CLL plasma can amplify NET release from healthy neutrophils through interleukin (IL)‐8 mediated priming effect [13]. Moreover, immature neutrophils during acute promyelocytic leukemia (APL) show impaired NET production, even after in vitro differentiation, whereas mature neutrophils display robust NET release and can induce platelet activation [14]. In addition, APL blasts can undergo NETosis by releasing intact chromatin into the extracellular space in response to various stimuli [15], thereby promoting APL‐associated coagulopathy, thrombin generation, fibrinolysis‐resistant fibrin clot deposition, and shortening plasma clotting time [16].

Based on the evidence that NETosis could play a pathogenetic role in certain hematological conditions, we screened elastase levels in various hematological malignancies, including lymphomas, plasma cell dyscrasias, and acute lymphoblastic leukemia, and we correlated these results with clinical and phenotypical features to identify potential prognostic biomarkers of disease severity and of differential diagnosis.

## 2 Materials and Methods

### 2.1 Patients

For elastase level assessment, a total of 111 patients and 42 healthy donors were recruited at the Hematology Unit of San Giovanni di Dio e Ruggi d'Aragona Hospital in Salerno, from January 2020 to December 2025, who were screened for any hematological malignancy according to current guidelines at the Hematology and Transplant Center, University Hospital “San Giovanni di Dio e Ruggi d'Aragona” of Salerno, Italy (Table S1). Patients were diagnosed with multiple myeloma (MM, *N* = 24), B‐cell lymphomas (NHL, *N* = 26), myelodysplastic syndromes (MDS, *N* = 16), CLL (*N* = 13), acute myeloid leukemia (AML, *N* = 11), acute lymphoblastic leukemia (ALL, *N* = 4), monoclonal gammopathy of undetermined significance (MGUS, *N* = 5), bone marrow failure syndromes (BMFS, *N* = 4), hemolytic anemia (*N* = 1), Hodgkin lymphoma (*N* = 1), CML (*N* = 1), or idiopathic myelofibrosis (IMF, *N* = 1). Age and sex‐matched HCs were recruited from donors at the Transfusion Medicine Unit, University Hospital “San Giovanni di Dio e Ruggi d'Aragona” of Salerno, Italy, and they were screened for any hematological and viral disorders before collection. Clinical characteristics are summarized in Table S1. PB samples were obtained from patients in ethylenediaminetetraacetic acid (EDTA) tubes and centrifuged at 2000 rpm for 20 min at room temperature (RT) for plasma collection.

For LDGs immunophenotyping, whole PB in EDTA tubes was drawn from a total of 33 consecutive subjects enrolled from January 2020 to Decembre 2025 who were screened for hematological malignancies at the Hematology and Transplant Center, University Hospital “San Giovanni di Dio e Ruggi d'Aragona” of Salerno, Italy (Table S2). After work‐up, patients received a diagnosis of IMF (*N* = 5), immune thrombocytopenia (*N* = 5), chronic or acute leukemias (*N* = 5), acquired aplastic anemia (AA) (*N* = 2), hemolytic anemia (*N* = 1), blastic plasmacytoid dendritic cell neoplasia (*N* = 1), plasma cell dyscrasias (*N* = 4), MDS (*N* = 3), NHL (*N* = 4), and leukocytosis of unknown origin (*N* = 2), and chemotherapy was administered as per international guidelines, where appropriate. A group of healthy subjects (*N* = 21) was used as a control, after ruling out the presence of cytopenia(s) or other inflammatory/pathological conditions as per medical screening.

All subjects in this study were enrolled after informed consent was obtained in accordance with the Declaration of Helsinki and protocols approved by our local Ethics Committee “Campania Sud” (Brusciano, Naples, Italy; prot./SCCE n. 24988).

### 2.2 Elastase Assay

Quantitative elastase measurement was carried out using the Human Neutrophil Elastase ELISA Kit (Abcam, ab270204, Cambridge, UK) following manufacturers' instructions. Briefly, plasma samples were diluted 1:50, incubated with Antibody Cocktail for 1 h at RT on a plate shaker, washed three times, and then TMB solution was added to each well. After incubation for 10 min in the dark, stop solution was added to each well, samples were incubated for 1 min, and then OD absorbance was read in a microplate reader at 450 nm (InfinitePro 200, Tecan Group Ltd., SW). The experiment was conducted in sets of two technical replicates.

### 2.3 Flow Cytometry

Neutrophil phenotype characterization was performed as previously described [1], by staining 50 μL of fresh heparinized whole PB with the following antibodies according to the manufacturers' instructions: CD56‐ECD, CD45‐Krome Orange, CD34‐APC AlexaFluor700, CD19‐PC5.5, CD11b‐PC7, CD3‐ECD, CD33‐APC, HLA‐DR‐FITC, CD15‐PE, CD14‐APC AlexaFluor750, and CD16‐Pacific Blue (all from Beckman Coulter, Milan, Italy). After 20 min incubation at RT, red cells lysis was carried out using IO Test Lysing Solution (Beckman Coulter), samples were washed twice with phosphate‐buffered saline (PBS) (Beckman Coulter), and then resuspended in 500 μL PBS for acquisition. A Navios/EX or a DxFlex cytometer (Beckman Coulter) was used for sample acquisition using the same PMT voltages, and at least 200 000 events were recorded. Instrument daily quality control was performed using Flow‐Check Pro Fluorospheres (Beckman Coulter). Post‐acquisition analysis was carried out using Navios EX Software v2.0, or Kaluza Analysis Flow Cytometry Software v2.1.1 (Beckman Coulter).

### 2.4 Gating Strategy

Cell populations were first identified based on linear parameters (forward scatter area, FSC‐A) and CD45 expression, cells were gated, and CD19 and CD56/CD3 expression was investigated as previously described [1]. On CD19^−^CD56^−^CD3^−^ cells, HLA‐DR^−^CD34^−^ population was further detected and studied for CD33 and CD15 expression. On CD33^+^CD15^−^ neutrophils (normal density granulocytes, NDGs), the maturation curve was described by CD16 and CD11b expression, and immature CD16^−^CD11b^−^, intermediate CD16^−/+^CD11b^+^, and mature CD16^+^CD11b^+^ neutrophil subsets were identified. On CD33^+^ cells, CD14 and CD15 expression was further investigated, and CD15^+^CD14^−^ low‐density granulocytes (LDGs) were identified. Maturation was investigated using CD16 versus CD11b expression, and CD15 versus CD16. Immature CD16^−^CD11b^−^, intermediate CD16^−/+^CD11b^+^, and mature CD16^+^CD11b^+^ LDGs were identified, as well as immature CD15^+^CD16^−^, intermediate CD15^+^CD16^dim^, and mature CD15^+^CD16^+^ LDGs were gated. Lymphocytes were divided into T (CD3 or CD5 or CD7, CD4, and CD8), B (CD19), and NK cell (CD56 and CD16) markers.

### 2.5 Statistical Analysis

Data were analyzed using Prism (v.11.0.0; GraphPad software, La Jolla, CA, USA). Cell populations were reported as percentages of positive cells. Parametric or non‐parametric data distribution was first tested using the D'Agostino & Pearson, Anderson‐Darling, Shapiro–Wilk, and Kolmogorov–Smirnov tests. Based on normality analysis results, Pearson or Spearman analysis was employed for studying correlations, or unpaired two‐tailed *t*‐ or non‐parametric Mann Whitney or Kolmogorov–Smirnov tests for two group comparison and Kruskal–Wallis test for three‐group comparison were performed with Dunn's test. ROC and area under the curve (AUC) analysis was also performed to determine sensitivity and specificity of each biomarker. A post hoc analysis was performed to calculate the power of our investigation, considering the number of patients and healthy subjects, and an alpha error of 0.05, and resulting in a power of 86%. RStudio (v.2022.07.1+554; RStudio software, Boston, MA, US) was employed for data visualization with correlograms using *corrplot* and *PerformanceAnalytics* packages. A *p* < 0.05 was considered statistically significant.

## 3 Results

### 3.1 Plasma Elastase Levels Are Increased in Hematological Malignancies

**FIGURE 1:**
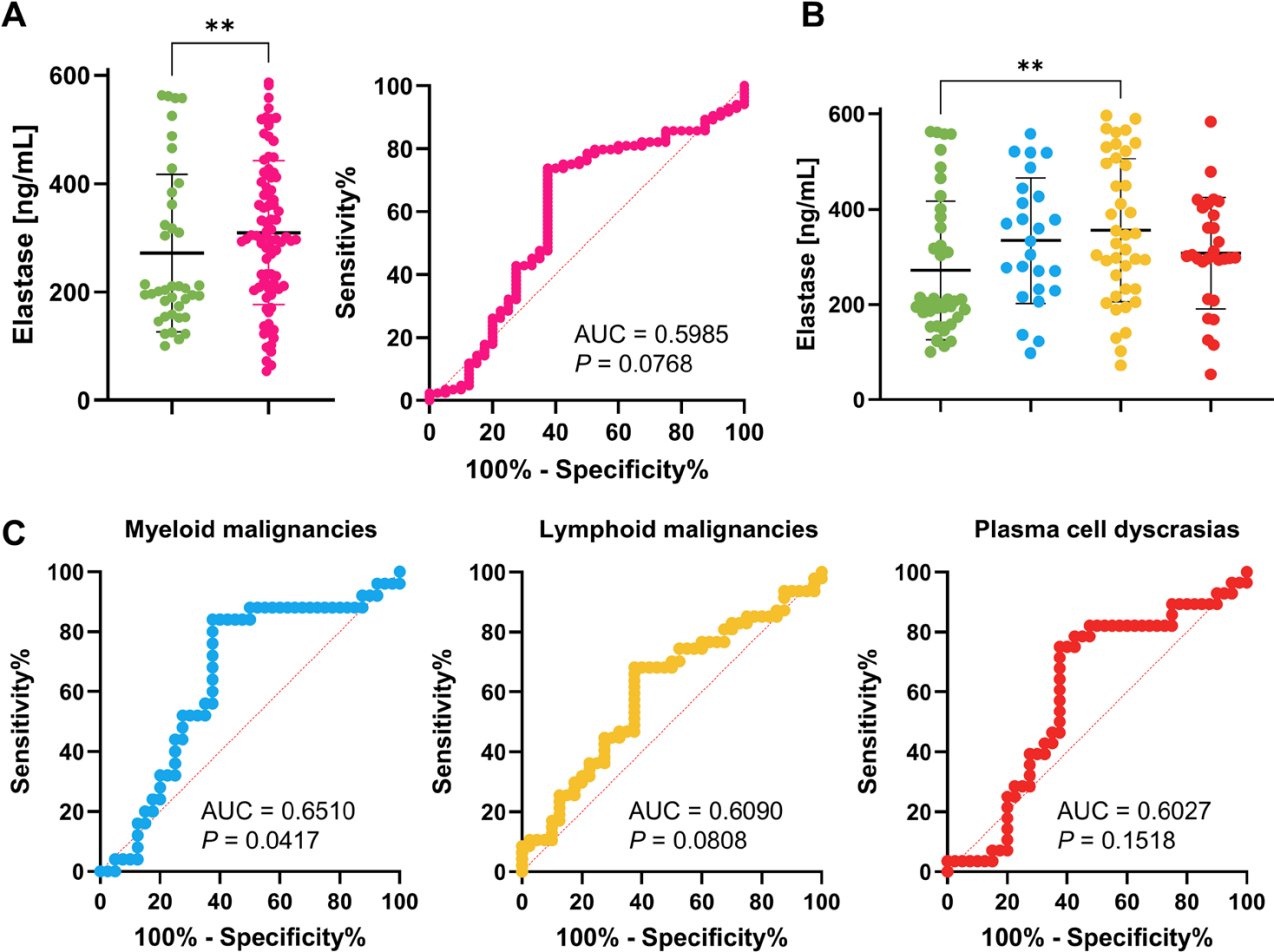
Plasma elastase levels in hematological conditions. (A) Plasma levels were compared between healthy controls (green dots) and patients with any hematological condition (purple dots), and ROC analysis was also performed. (B) Patients were divided in myeloid (blue dots), lymphoid (yellow dots), or plasma cell malignancy (red dots), and plasma elastase levels were compared between groups and HC. (C) A ROC curve analysis for investigating the diagnostic power was performed for each group. AUC, area under the curve. A *p* < 0.05 was considered statistically significant. *, *p* < 0.05; **, *p* < 0.01.

First, to explore the diagnostic potential of plasma elastase, circulating levels were compared between HC and hematological patients, regardless of diagnosis (Figure 1A). Plasma elastase levels were significantly increased in hematological patients (mean ± SD, 309.2 ± 132.6 ng/mL vs. 271.6 ± 145.4 ng/mL, HC vs. patients; *p* = 0.0016), and the ROC analysis showed a potential good specificity and an AUC of 0.5985 (*p* = 0.0768). In particular, a cut‐off elastase value of < 300 ng/mL displayed a sensitivity of 50% (95% confidence interval [CI], 39.5%–60.5%) and a specificity of 62.5% (95% CI, 47%–75.8%; likelihood ratio, 1.3). Therefore, we chose this value for further subgroup analysis. Moreover, diseases with *N* = 1 were not included in further analyses.

Next, to identify the hematological malignancy with the highest elastase variation, patients were divided in three groups: myeloid or lymphoid neoplasms, and plasma cell dyscrasias (Figure 1B). Plasma elastase tended to be increased in myeloid neoplasms compared to HC (mean ± SD, 334.0 ± 131.7 vs. 271.6 ± 145.4 ng/mL, respectively; *p* = 0.0528), as well as in patients with plasma cell dyscrasias (mean ± SD, 307.6 ± 116.9 ng/mL) compared to HC (*p* = 0.1557), while patients with lymphoid neoplasms had significantly higher levels compared to HC (mean ± SD, 356.0 ± 131.7 ng/mL; *p* = 0.0054). A ROC analysis was conducted for an initial screening to investigate sensitivity and specificity of plasma elastase as a diagnostic marker of myeloid, lymphoid, and plasma cell neoplasia (Figure 1C). In patients with myeloid malignancies, the AUC was 0.6510 (*p* = 0.0417), and at the cut‐off value, sensitivity was 56% (95% CI, 37.1%–73.3%) and specificity was 62.5% (95% CI, 47%–75.8%; likelihood ratio, 1.5). Similarly, in patients with plasma cell dyscrasias, the AUC was 0.6027 (*p* = 0.1518), and using the cut‐off value of 300 ng/mL, sensitivity was 50% (95% CI, 32.6%–67.4%) and specificity was 62.5% (95% CI, 47%–75.8%; likelihood ratio, 1.3). In lymphoid neoplasms, the AUC was 0.6090 (*p* = 0.0808), and using the cut‐off value of 300 ng/mL, sensitivity was 48.9% (95% CI, 35.3%–62.8%) and specificity was 62.5% (95% CI, 47%–75.8%; likelihood ratio, 1.3).

### 3.2 Plasma Elastase as a Potential Biomarker for Differential Diagnosis

**FIGURE 2:**
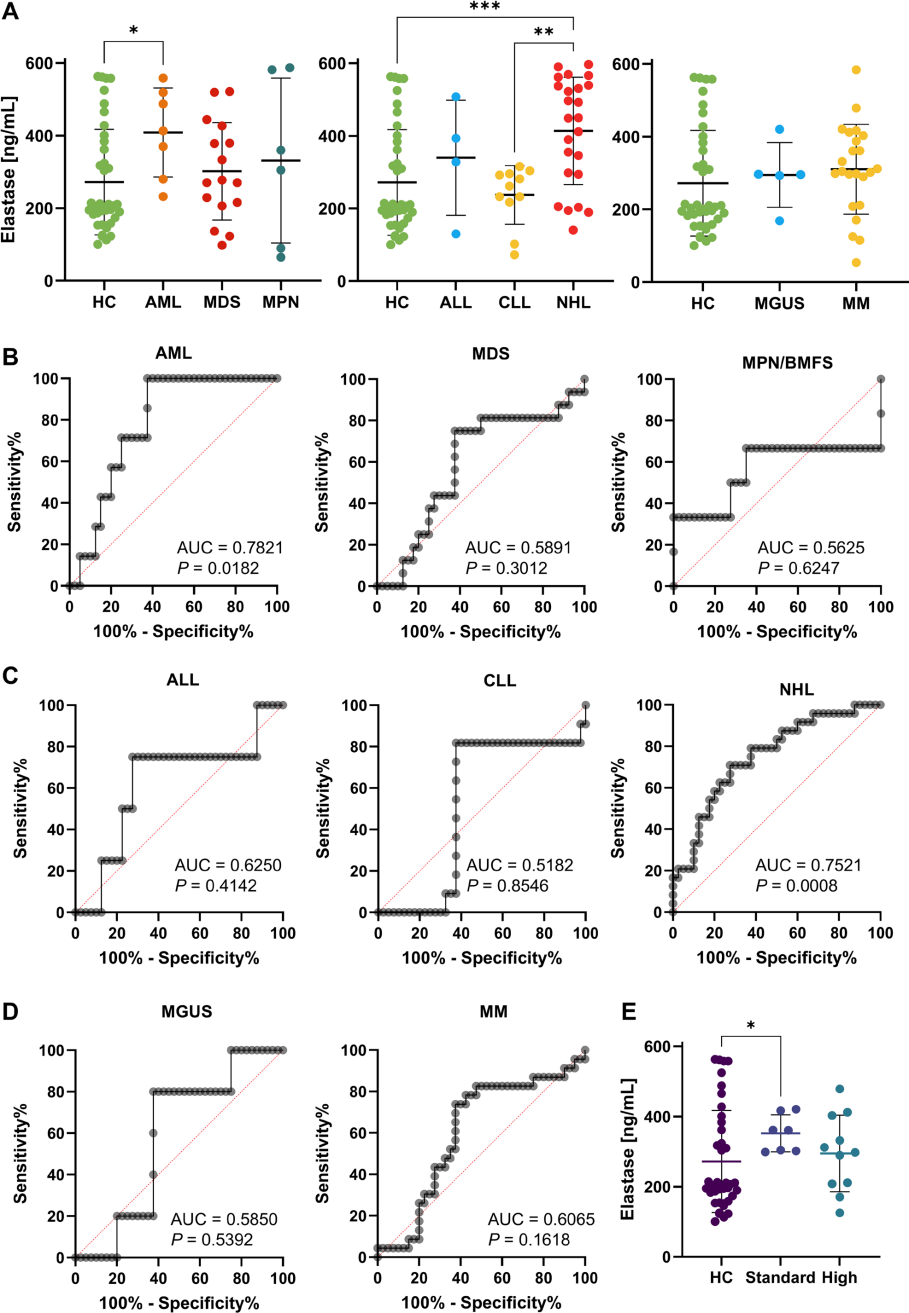
Plasma elastase levels based on hematological disease. (A) Plasma levels were compared between healthy controls (green dots) and patients with each hematological condition, and a ROC analysis was also performed for (B) myeloid and (C) lymphoid neoplasm subtypes and for (D) plasma cell dyscrasias. (E) Plasma levels were also compared between MM patients with standard or high risk genetic. ALL, acute lymphoblastic leukemia; AML, acute myeloid leukemia; AUC, area under the curve; CLL, chronic lymphocytic leukemia; MDS, myelodysplastic syndromes; MGUS, monoclonal gammopathy of uncertain significance; MM, multiple myeloma; MPN, myeloproliferative neoplasms; NHL, non‐Hodgkin lymphomas. A *p* < 0.05 was considered statistically significant. *, *p* < 0.05; **, *p* < 0.01; ***, *p* < 0.001.

Subsequently, whether to investigate the potential role of plasma elastase as a biomarker for differential diagnosis between hematological malignancies, patients were divided according to their diagnosis, and plasma levels were compared between groups (Figure 2). In detail, patients with myeloid malignancies were divided in AML, MDS, and MPN (also including BMFS), and plasma elastase levels were significantly increased in AML compared to HC (mean ± SD, 408.3 ± 122.5 vs. 271.6 ± 145.4 ng/mL, respectively; *p* = 0.0204) and tended to be decreased in MDS patients (mean ± SD, 301.7 ± 134.0 ng/mL) compared to AML (*p* = 0.1384), while no significant differences were observed in MPN subjects (mean ± SD, 331.2 ± 227.4 ng/mL; *p* = 0.4750) compared to HC. ROC analysis showed a significant AUC for AML discrimination (AUC = 0.7821; *p* = 0.0182), and using the cut‐off value of 300 ng/mL, sensitivity 71.4% (95% CI, 35.6%–94.9%) and specificity was 65% (95% CI, 49.5%–77.9%; likelihood ratio, 2.04) (Figure 2B).

For lymphoid malignancies, patients were divided in ALL, CLL, and NHL group, and plasma elastase levels were compared between groups and HC. CLL patients displayed the lowest plasma levels (mean ± SD, 236.9 ± 80.8 ng/mL) compared to NHL subjects (mean ± SD, 413.6 ± 147.8 ng/mL; *p* = 0.0082), while plasma levels were significantly augmented in NHL compared to HC (mean ± SD, 413.6 ± 147.8 vs. 271.6 + 145.4 ng/mL, respectively; *p* = 0.0004). ROC analysis showed a significant AUC for NHL discrimination (AUC = 0.7521; *p* = 0.0008), and using the cut‐off value of 300 ng/mL, sensitivity 70.8% (95% CI, 50.8%–85.1%) and specificity was 62.5% (95% CI, 47%–75.8%; likelihood ratio, 1.9) (Figure 2C).

For plasma cell dyscrasias, patients were divided in MGUS and MM groups, and levels were compared (Figure 2A). No significant variations were observed in MM patients (mean ± SD, 310.4 ± 123.6 ng/mL) compared to HC (*p* = 0.1356) or MGUS subjects (mean ± SD, 294.7 ± 88.9 ng/mL; *p* = 0.7031), because of the small number of patients in this group. ROC analysis showed no significant AUC for MGUS discrimination (AUC = 0.5850; *p* = 0.5392), and a potential significant AUC for MM discrimination (AUC = 0.6065; *p* = 0.1618) was observed (Figure 2D). Because of this possible association with plasma cell dyscrasias, MM patients were further divided based on genetic risk: R‐ISS standard (including normal karyotype, deletion of chromosome 17, or 17p‐, translocation of chromosomes 14 and 16, or *t*[14;16], and translocation of chromosomes 4 and 14, or *t*[4;14]); and high‐risk genetic (EMMA risk stratification including del[17p] > 20%, *TP53* mutation, biallelic del1p32, 1q gain and monoallelic del1p32, *t*[4;14] or *t*[14;16] or *t*[14;20] and either 1q gain or monoallelic del1p32) (Figure 2E). Although the number of patients in each group was small, high‐risk MM patients tended to display lower plasma elastase levels (mean ± SD, 294.5 ± 109.3 ng/mL) compared to standard‐risk MM (*p* = 0.3861), who showed higher elastase levels compared to HC (mean ± SD, 351.9 ± 52.8 vs. 371.6 ± 145.4 ng/mL, respectively; *p* = 0.0404).

### 3.3 Correlations With Clinical Features and Outcomes

**FIGURE 3:**
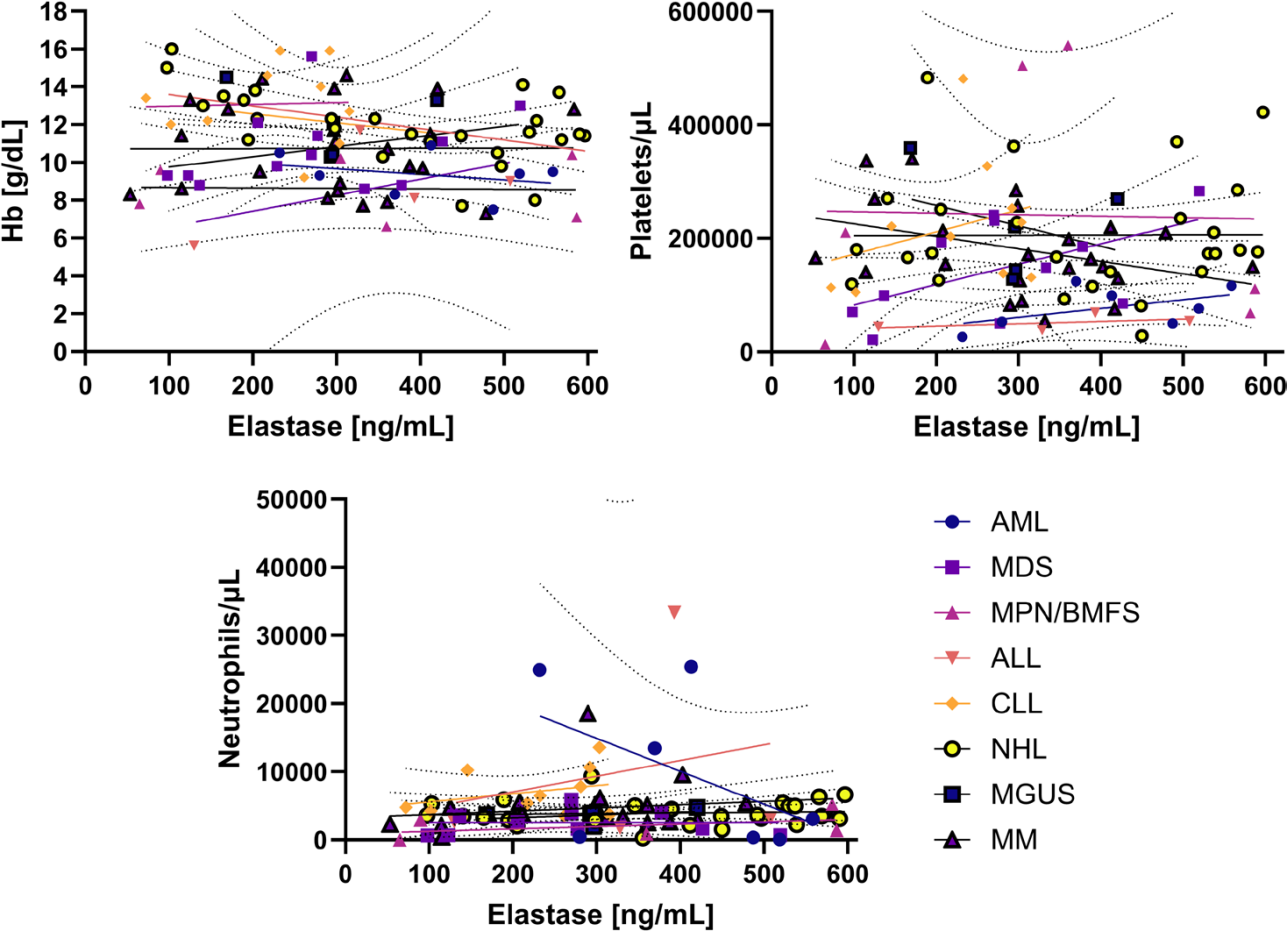
Correlations with complete blood counts. Plasma elastase levels were compared to hemoglobin levels, absolute neutrophil and platelet counts by Pearson correlation analysis, also by dividing patients according to their diagnosis. ALL, acute lymphoblastic leukemia; AML, acute myeloid leukemia; CLL, chronic lymphocytic leukemia; MDS, myelodysplastic syndromes; MGUS, monoclonal gammopathy of uncertain significance; MM, multiple myeloma; MPN, myeloproliferative neoplasms; NHL, non‐Hodgkin lymphomas. Dashed lines, 95% confidential interval.

Next, plasma elastase levels were correlated with complete blood counts (CBCs) to exclude the influence of blood counts on plasma elastase variations (Figure 3). Patients were also divided based on their diagnosis, and plasma elastase levels were compared to hemoglobin levels, platelet count, and absolute neutrophil count. For platelets and absolute neutrophil counts, no significant associations were found for any hematological malignancy (all *p* > 0.05), while for hemoglobin levels, a significant negative correlation was observed in NHL patients (*r* = −0.5337; *p* = 0.0050).

**FIGURE 4:**
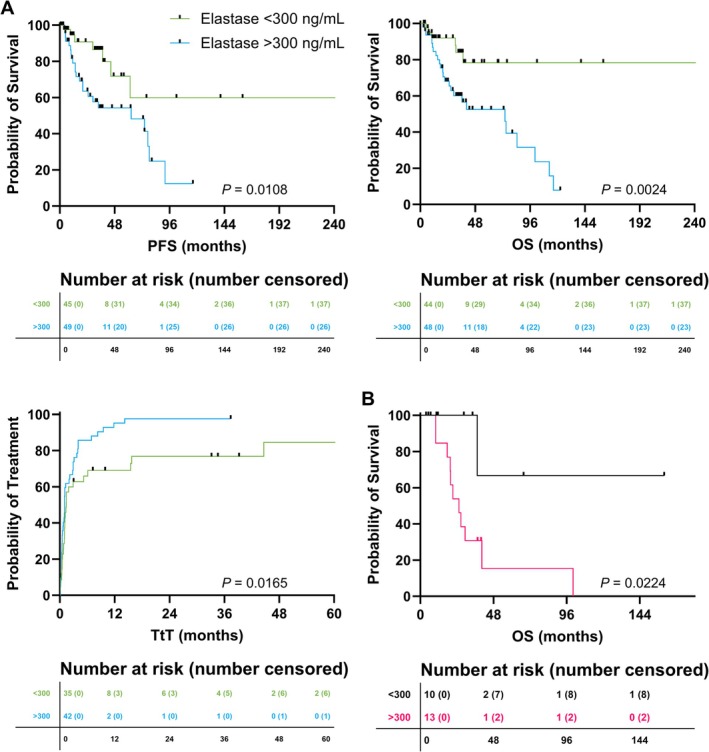
Prognostic potential of plasma elastase levels. Patients were divided based on plasma neutrophil elastase levels in two groups using as a cut‐off value a 300 ng/mL concentration: Patients with low levels (< 300 ng/mL) and those with increased levels (> 300 ng/mL). Clinical outcomes, such as overall survival (OS), progression‐free survival (PFS), and time‐to‐treatment (TtT) were compared between groups in (A) the entire cohort or (B) in multiple myeloma patients. Number of censored subjects is also reported.

Subsequently, to assess the prognostic significance of plasma elastase levels, patients were divided using the cut‐off value of 300 ng/mL, which has shown good sensitivity and specificity across hematological diseases, and clinical outcomes were compared between groups (Figure 4). Patients with low elastase levels displayed a significantly longer progression‐free survival (PFS) compared to those with higher levels (median PFS, not reached vs. 62.5 months; 95% CI, N.E. and 20–78.3 months, respectively; *p* = 0.0108), as well as a longer overall survival (OS; median OS, 272.1 months vs. 74 months; 95% CI, N.E. and 25.3–100.3 months, respectively; *p* = 0.0024) (Figure 4A). Moreover, patients with low elastase levels showed a longer time‐to‐treatment (TtT) compared to those with higher levels (median TtT, 1.37 months vs. 1.02 months; 95% CI, 1.0–5.2 and 0.6–2 months, respectively; *p* = 0.0165). Because of the association with MM, we conducted a subanalysis by dividing MM patients based on elastase values < 300 or > 300 ng/mL (Figure 4B). Patients with high elastase values showed a longer PFS (median PFS, 387.5 months vs. 37.4 months; 95% CI, 311.9–416.6 months and 9.2‐N.E., > 300 and < 300 ng/mL, respectively; *p* = 0.0003), while a shorter OS (median OS, 25.3 months vs. not reached; 95% CI, 17.6–40.4 months and N.E., > 300 and < 300 ng/mL, respectively; *p* = 0.0224) (Figure 4B).

### 3.4 Granulocyte Terminal Differentiation Is Altered in Hematological Disorders

**FIGURE 5:**
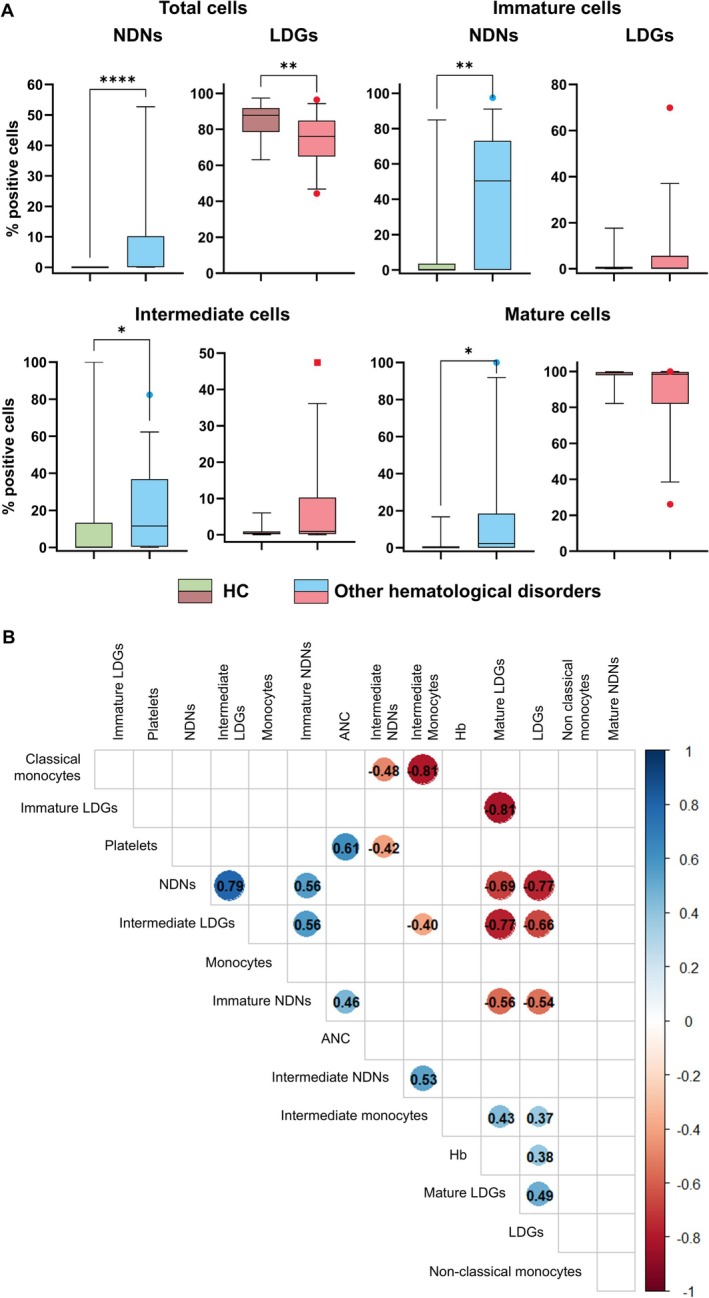
Granulocyte subset frequency distribution and correlations in benign and malignant hematological conditions. (A) Percentages of total normal density neutrophils (NDNs), low‐density granulocytes (LDGs), and their subsets were calculated for patients with various benign and malignant hematological conditions, and compared to healthy subjects. Data are shown as median with IQR. Kruskal–Wallis test was performed. *p < 0.05; **p < 0.01. (B) Circulating NDN and LDG subsets were also correlated. Values range between −1 (red) and +1 (blue). Related *p* values are reported in Figure S1.

To investigate whether elastase levels could mirror neutrophil differentiation status, rather than their circulating levels, granulocyte differentiation and subset distribution, including NDN and LDG maturation stages, were assessed in a cohort of 18 subjects with various hematological conditions. NDN levels were significantly higher in hematological patients compared to healthy subjects (median with IQR, 0.2%, 0.03%–7.34% vs. 0%, 0%–0.02%, respectively; *p* < 0.0001) (Figure 5A), while LDGs were significantly lower in patients compared to healthy individuals (median with IQR, 75.4%, 59.7%–85.8% vs. 87.8%, 79%–91.7%, respectively; *p* = 0.0031). In particular, all NDN subsets were highly represented in hematological patients compared to HC: immature NDNs, median with IQR, 50.4%, 0%–72.2%, versus 0%, respectively, *p* = 0.0027; intermediate NDNs, median with IQR, 11.6%, 0.9%–34.5%, versus 0%, 0%–9.1%, respectively, *p* = 0.0174; and mature NDNs, median with IQR, 2.3%, 0%–17.5%, versus 0%, respectively, *p* = 0.0115. Conversely, only intermediate LDGs tended to be increased in hematological patients compared to healthy subjects (median with IQR, 0.94%, 0.27%–8.3% vs. 0.4%, 0.13%–0.89%, respectively; *p* = 0.0618).

Next, granulocyte subset frequencies were correlated with clinical parameters, such as hemoglobin levels, absolute neutrophil count, platelet count, and percentage of circulating leukemic cells or hematopoietic stem cells (HSC). Intermediate NDNs were negatively correlated with platelet count (*r* = −0.42; *p* = 0.0244), while LDGs were positively associated with hemoglobin levels (*r* = 0.38; *p* = 0.0359) (Figure 5B). Classical monocytes were inversely related to intermediate monocytes (*r* = −0.81) and intermediate NDNs (*r* = −0.48), while intermediate monocytes were positively correlated with mature LDGs (*r* = 0.43) and total LDG frequencies (*r* = 0.37). Immature LDG forms were negatively associated with mature LDGs (*r* = −0.81), while intermediate LDGs were positively related to immature NDNs (*r* = 0.56), and negatively associated with intermediate monocytes (*r* = −0.40), mature LDGs (*r* = −0.77), and total LDGs (*r* = −0.66) (all *p* < 0.05). Moreover, total NDNs were positively associated with immature NDN forms (*r* = 0.56) and intermediate LDGs (*r* = 0.79), while negatively related to mature LDGs (*r* = −0.69) and total LDGs (*r* = −0.77).

### 3.5 Cross‐Validation of Plasma Elastase in Large‐Scale Proteomics Studies

Finally, to validate our preliminary results of altered plasma neutrophil elastase levels in hematological malignancies, we searched for publicly available datasets of large‐scale proteomics studies conducted in various hematological malignancies. An aptamer‐based high‐throughput proteomics analysis in acquired AA, an immune‐mediated BMFS, was used to investigate NET‐associated plasma proteins, including proteinase 3 (PRTN3), lactoferrin (LTF), transketolase (TKT), elastase, MPO, and lysozyme [1]. PRTN3, LTF, and TKT were significantly increased in the plasma of healthy individuals compared to AA at diagnosis (*p* < 0.0001, *p* = 0.0223, and *p* < 0.0001, respectively), and their levels restored in AA responders after 6 months of immunosuppressive therapies (Table S3). Conversely, their levels remained lower compared to HCs in non‐responders after 6 months of therapies (*p* = 0.0002, *p* = 0.0019, and *p* < 0.0001, respectively). Similarly, MPO and elastase were higher in the plasma of healthy individuals compared to AA patients at diagnosis (*p* = 0.0017 and *p* < 0.0001, respectively), especially at diagnosis in those who did not respond to therapies at the six‐month time point (*p* < 0.0001 and *p* = 0.0136, respectively).

A dataset of aptamer‐based proteomics analysis of patients with relapsed/refractory AML was retrieved to confirm plasma elastase levels alterations in this condition [17]. In this dataset, elastase was only slightly lower in the plasma of AML patients compared to healthy subjects (log_2_ fold‐change, −0.71; *p* = 0.017), although this difference was more marked in the bone marrow (BM) plasma (log_2_ fold‐change, −1.25; *p* = 0.038). Finally, another aptamer‐based large‐scale proteomic dataset of patients with Adult T‐cell leukemia/lymphoma (ATL), a human T‐cell leukemia virus type 1 (HTLV‐1)‐associated T‐cell malignancy with generally poor prognosis, has been retrieved (Table S4) [2]. Notably, elastase levels were comparable between asymptomatic carriers (AC) and patients with ATL (*p* = 0.596), while they significantly changed during remission compared to carriers (*t*‐test, −2.7, remission vs. AC; *p* = 0.032) or ATL at diagnosis (*t*‐test, −2.3, remission vs. ATL; *p* = 0.0297) (Table S2). Conversely, MPO levels were significantly different in AC versus ATL (*t*‐test, 3.68; *p* = 0.0006), and also in responders compared to ATL patients at diagnosis (*t*‐test, −4.5, remission vs. ATL; *p* = 0.0002).

## 4 Discussion

NETs are extracellular web‐like structures of decondensed chromatin and mitochondrial DNA on which cytosolic and granule proteins are assembled [8]. NET composition varies in health and diseases [18]; however, some components are often found across different diseases, and MPO is one of the main elements, which is also used for the detection and quantification of NETs [8]. In autoimmune disorders, NETs can be a source of self‐antigens, and components are detected in the blood or in the synovial fluid of patients in response to type I interferons (IFNs) and ROS signaling pathways [18]. In addition, NETs can directly drive type I IFN production by plasmacytoid dendritic cells in a positive feedback loop, contributing to tissue damage [19]. Other proteins frequently present in NETs are alarmins of the S100A family, including the heterodimer calprotectin, or PRTN3, LTF, TKT, and elastase [20]. In this report, we showed the potential role of circulating plasma elastase as a diagnostic and prognostic biomarker of various hematological malignancies.

Neutrophil elastase is a serine protease expressed in neutrophils and stored in their azurophilic granules, and is linked to the regulation of inflammation, bacterial infection progression, mucus secretion, cytokine production, and immune response modulation [1, 21, 22]. Neutrophil elastase can negatively influence tumor suppressor activities, leading to tumor growth and metastasis, also by inducing angiogenesis [21, 22]. Indeed, increased circulating and tissue elastase levels are associated with worse outcomes in several cancers [22]. Moreover, we have previously shown that plasma elastase is significantly decreased in MDS patients compared to AML and healthy subjects, suggesting reduced granulocyte functions, which could predispose to infectious disease susceptibility and sustain proinflammatory immune responses [1]. According to our previous findings, circulating plasma elastase tended to be reduced in MDS compared to AML, displaying a potential good sensitivity and a high specificity for differential diagnosis with an AUC of 0.7232 (AML vs. MDS, *p* = 0.0948). Conversely, plasma elastase was found increased in AML and NHL compared to healthy subjects, showing a good sensitivity as a diagnostic marker, and for differential diagnosis between CLL and NHL (AUC, 0.8106, *p* = 0.0036), with a sensitivity of 70.8% and a specificity of 81.8% at a cut‐off value of 300 ng/mL (likelihood ratio, 3.9). These findings in AML are in accordance with previous studies showing that elastase activity can be detected in almost all patients with acute non‐lymphoblastic leukemias, especially promyelocytic leukemia, while not in ALL [23]. Indeed, our ALL patients did not display elevated plasma elastase levels, although our cohort was limited. However, plasma elastase levels could greatly vary in AML subtypes, ranging from extremely low or low levels in monocytic leukemia (M5) and leukemia without maturation (M1) to extremely high in promyelocytic leukemia (M3) [24], likely because of the abundance of granulated blasts. In AML cells, cell‐surface elastase expression is promoted by the chemokine stromal cell‐derived factor‐1, and elastase inhibition reduces leukemic cell migration and egress from the BM, and also impairs hematopoietic stem and progenitor cell homing to the marrow niche after transplantation in mouse models [25]. Therefore, we supported the evidence of a pathogenic role of plasma elastase in AML, and we also suggested that plasma elastase levels could be altered in MDS and could be influenced by circulating frequency of blasts, as high‐risk MDS could display higher plasma elastase levels compared to low‐risk MDS (*p* = 0.1680), although no conclusions can be made because of the small number of patients in each clinical category. However, our results supported the findings that plasma elastase is greatly related to the frequency of leukemic cells [23, 24].

In CLL, neutrophils are more susceptible to release NETs compared to healthy subjects, independently from neutrophil expression of elastase, MPO, or reactive oxygen species production [13]. This higher NET release is likely due to higher plasma levels of IL‐8, rather than increased elastase and MPO expression, suggesting that this altered chronic microenvironment also influences neutrophil activities. Conversely, CLL cells lack elastase expression, as this enzyme can degrade an alternative ligand of VLA‐4 (CD49d expressed on neoplastic cells), the Elastin MIcrofibriLINterfacer‐1 (EMILIN‐1), an extracellular matrix multi‐domain glycoprotein [26]. Indeed, CLL cells highly express EMILIN‐1, while not elastase. Therefore, reduced plasma elastase levels could favor CLL migration and tissue invasion, through integrin‐extracellular matrix interaction. Conversely, lymphoma cells can express elastase upon IL‐6, IL‐13, and metalloproteinase 9, and can recruit it at their surface via ICAM‐1. The presence of elastase on lymphoma cells is involved in aggressive behavior of neoplastic cells, with increased ability of invasiveness [27]. According to these findings, we showed that plasma elastase was significantly increased in NHL compared to healthy subjects and CLL.

In MM, plasma cells are derived from B cells, which physiologically lack neutrophil elastase and PRTN3, while they still maintain antigen‐presenting functions. Neoplastic plasma cells can uptake these molecules and present them as PR1, a nonameric peptide derived from PRTN3 and neutrophil elastase, with a peak in cross‐presentation at 24 and 6 h, respectively; however, PR1 presentation increases cytotoxic T cell‐mediated killing of neoplastic plasma cells [28]. Moreover, myeloma cells are able to induce citrullination of histone H3, triggering NETs formation, and their release from neutrophils [29]. Higher NET levels are found in the plasma of MM and correlate with disease severity [30], and single‐cell dataset analysis reveals a NET‐related prognostic signature for MM, with encouraging AUC for survival prediction [13]. In addition, plasma elastase and MPO levels are increased in MM patients with extramedullary disease compared to those without extra BM localization [31]. In our study, we showed that plasma elastase levels could be increased in standard‐risk MM patients, supporting the pathogenic role of NETs in MM. Moreover, high levels were associated with worse outcomes, likely because of more aggressive diseases, such as those with extramedullary involvement.

BMFS are a heterogeneous group of benign hematological diseases characterized by uni‐ or multi‐lineage marrow and/or PB cytopenia(s) [32, 33, 34, 35]. In constitutional syndromes, inherited germline mutations occurring in the HSC compartment lead to marrow failure; while acquired syndromes are caused by extrinsic direct and indirect damages on the HSC pool due to chemical agents, drugs, viruses, or other unknown injuries [32] leading to an immune‐mediated BM destruction [33, 34, 35]. In BMFS, NET‐related components, such as circulating S100A8, are decreased, while they increase in responders to therapies [36]. From reported aptamer‐based proteomics analysis of plasma obtained from AA patients [32], other NET‐related proteins, such as PRTN3, LTF, and TKT, are significantly increased in the plasma of healthy individuals compared to AA at diagnosis; while no differences are found between HCs and AA responders after 6 months of therapy [32]. Similarly, MPO and elastase are higher in the plasma of healthy individuals compared to non‐responders. Therefore, decreased levels of those proteins in the plasma of AA could simply mirror reduced marrow cellularity and diminished neutrophils, as a surrogate marker of the number of circulating cells. For this reason, correlation analysis with CBCs was performed, to exclude the possibility that plasma elastase levels were influenced by the number of circulating PB cells. As expected, no significant associations were observed between plasma elastase and hemoglobin (except in NHL), absolute neutrophil count, and platelets count in all investigated hematological malignancies. Our results supported the hypothesis that altered plasma neutrophil elastase could be a marker of impaired granulocyte function, rather than a simple assay for residual neutrophil count quantification. As a proof‐of‐concept of this hypothesis, we showed that NDN and LDG subsets were significantly impaired in the PB of patients with any hematological conditions, suggesting that granulocyte subset perturbations could not only be an epiphenomenon, while could sustain emergency hemopoiesis and pro‐inflammatory responses, favoring disease progression and evolution, and circulating neutrophil elastase could be a marker of chronic inflammation during hematological malignancies. Indeed, using a cut‐off value of 300 ng/mL, hematological conditions could be discriminated with good sensitivity and specificity, especially for differential diagnosis between MDS and AML, or CLL and other NHL. Moreover, patients with low elastase levels could experience better outcomes compared to those subjects with increased levels, supporting an additional prognostic role of this minimally invasive biomarker.

Finally, cross‐validation using large‐scale aptamer‐based proteomics profiling confirmed that plasma elastase levels differed between healthy and hematological malignancies, with some differences across clinical entities. Indeed, neutrophil elastase was altered in AML, while substantial variations were observed in ATL, especially in those who achieved a complete remission. Therefore, these results supported the potential use of plasma elastase as an additional prognostic tool for differential diagnosis between hematological malignancies, and also as a prognostic marker of responsiveness to therapies.

Our study has several limitations: (i) the short follow‐up period that could not allow to definitely assess the prognostic role of plasma elastase; (ii) the small number of subjects for certain clinical entities, that could increase the risk of type II errors (false negatives), especially for clinically heterogenous diseases. Therefore, additional statistically significant differences could not be excluded, especially in MGUS and MDS subtypes. (iii) The lack of sequential measurements and immunophenotyping for the entire cohort for a comparison before and after therapies; (iv) additional functional in vitro analyses to confirm the proinflammatory role of elastase in hematological conditions; and (v) lack of a priori power analysis and of a minimum detectable effect calculation.

In conclusion, we widely screened plasma elastase levels even in a small cohort of hematological patients, and we also analyzed distributions and perturbations of LDG and NDN subsets, showing decreased levels of plasma elastase in certain malignancies, such as MDS and CLL, while highly increased in more aggressive diseases, like AML and NHL, thus proposing this biomarker as an additional diagnostic tool of differential diagnosis. Moreover, we indicated a cut‐off value of 300 ng/mL to discriminate between low‐ and high‐risk diseases, as patients with high plasma elastase levels showed worse outcomes, regardless of their diagnosis. In addition, we also support a potential clinical utility of neutrophil subset immunophenotyping, together with the standard CD16 versus CD11b maturation curve, as an additional flow cytometry marker of hematopoietic efficiency. However, further validation on a larger cohort is needed to confirm our results.

## Author Contributions

Conceptualization: V.G. and C.S. Data collection: P.S. A.M.D.C., and V.G. Methodology: P.S., A.M.D.C., A.B., M.G., F.P., B.C., and V.G. Clinical data: A.M.D.C., B.S., D.D.N., L.M., L.P., M.C.M., D.M., M.D., B.C., and V.G. Data analysis: P.S. and V.G. Writing – original draft preparation: P.S. Writing – review and editing. V.G. and C.S. All authors have read and agreed to the published version of the manuscript.

## Funding

This research was supported by the Aplastic Anemia & MDS International Foundation, grant cycle 2020 (Award ID, 724865). This research was also supported by the Intramural Program of the Department of Medicine, Surgery and Dentistry, University of Salerno, Italy (funding no. 300397FRB22SELLE and 300397EST21SELLE_01).

## Ethics Statement

Protocol approved by local ethic committee (Ethics Committee “Campania Sud,” Brusciano, Naples, Italy; prot./SCCE n. 24988).

## Consent

Patients received informed consent obtained in accordance with the Declaration of Helsinki (World Medical Association 2013) and protocols approved by the local ethic committee (Ethics Committee “Campania Sud,” Brusciano, Naples, Italy; prot./SCCE n. 24988).

## Conflicts of Interest

The authors declare no conflicts of interest.

## Supporting information


**Figure S1:** Correlation matrix used to build correlograms in Figure 5B.

**Table S1:** Patients' characteristics of the cohort for plasma elastase measurement.
**Table S2:** Patients' characteristics of the cohort for neutrophil subset immunophenotyping.
**Table S3:** Circulating levels of reported NET‐related proteins in the plasma of AA and HC [1].
**Table S4:** Circulating levels of reported NET‐related proteins in asymptomatic HLTV‐1 carriers (AC) and patients with Adult T‐cell leukemia/lymphoma (ATL) [2].

## Data Availability

The data that support the findings of this study are available from the corresponding author upon reasonable request.
