# Supplementary figures and images for: Plasma Elastase Screening in Hematological Disease Reveals Its Potential as a Diagnostic and Prognostic Biomarker in Hematological Malignancies
Source: Int J Lab Hematol. 2026 Mar 12;48(4):795–806. doi: 10.1111/ijlh.70090 (PMC13357920; doi:10.1111/ijlh.70090)

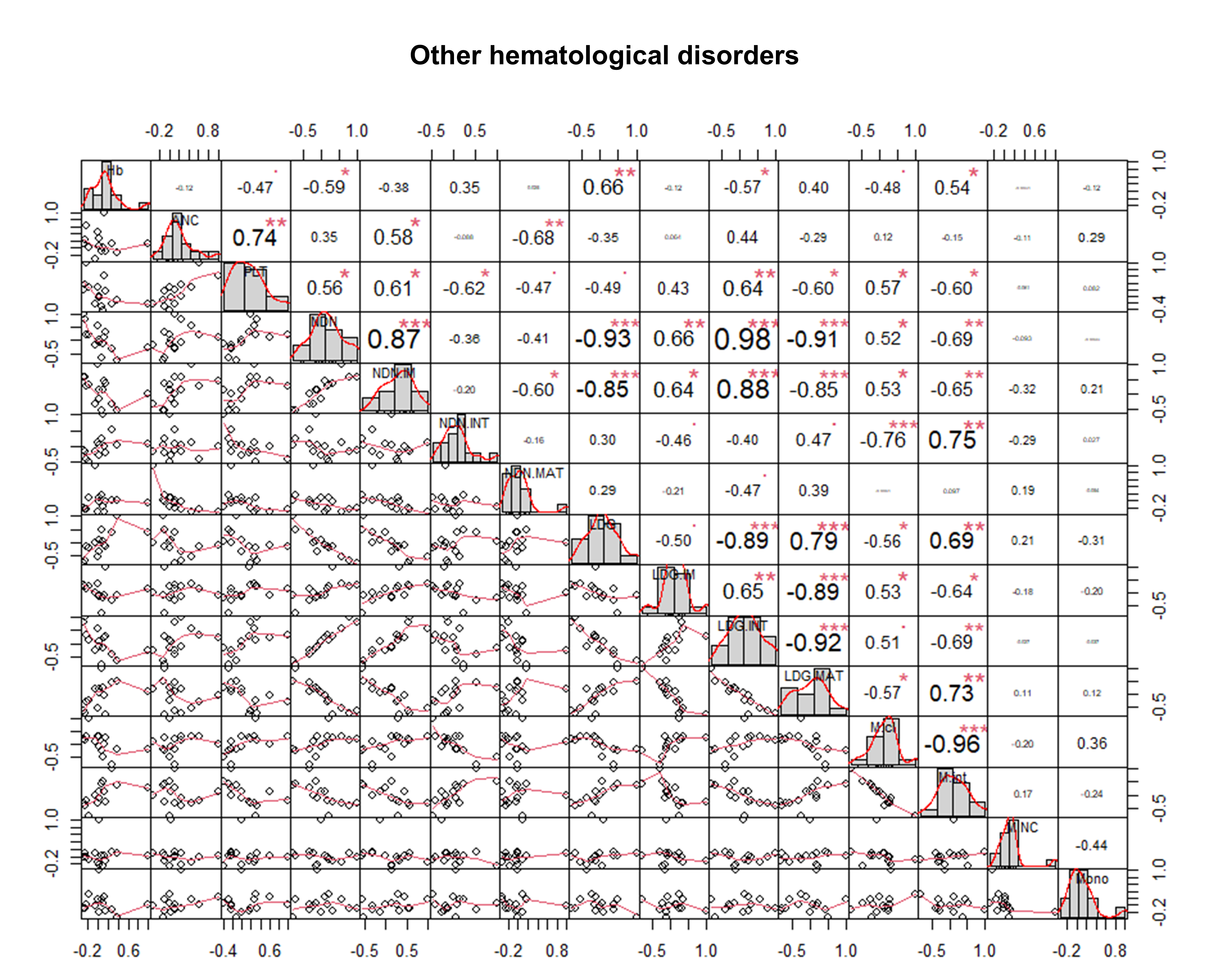

Supplement: Supplementary file 1 — Figure S1: Correlation matrix used to build correlograms in Figure 5B. [file IJLH-48-795-s001.tif]
