# Supplementary material for: Plasma Elastase Screening in Hematological Disease Reveals Its Potential as a Diagnostic and Prognostic Biomarker in Hematological Malignancies
Source: Int J Lab Hematol. 2026 Mar 12;48(4):795–806. doi: 10.1111/ijlh.70090 (PMC13357920; doi:10.1111/ijlh.70090)
Supplement: Supplementary file 2 — Table S1: Patients' characteristics of the cohort for plasma elastase measurement. Table S2: Patients' characteristics of the cohort for neutrophil subset immunophenotyping. Table S3: Circulating levels of reported NET‐related proteins in the plasma of AA and HC [1]. Table S4: Circulating levels of reported NET‐related proteins in asymptomatic HLTV‐1 carriers (AC) and patients with Adult T‐cell leukemia/lymphoma (ATL) [2]. [file IJLH-48-795-s002.docx]

**Supplementary Tables**

**Table S1. Patients’ characteristics of the cohort for plasma elastase measurement.**

| **Characteristics** | **Healthy subjects**  **N = 42** | **Patients**  **N = 111** |
| --- | --- | --- |
| Mean age, years old (range) | 62 (30-86) | 64 (24-90) |
| Sex (M/F) | 22/20 | 48/60 |
| 2022 WHO classification, n (%)  Multiple myeloma  MGUS  Myelofibrosis  Myelodysplastic syndromes  Non-Hodgkin lymphomas  Chronic myeloid leukemia  Acute myeloid leukemia  Chronic lymphocytic leukemia  Acute lymphoblastic leukemia  Hodgkin lymphoma  Bone marrow failure syndromes  Others | - | 24 (20)  5 (4)  1 (1)  16 (14)  27 (24)  1 (1)  11 (10)  12 (11)  4 (4)  1 (1)  4 (4)  2 (2) |
| Mean Hb, g/dL (range) | 11 (7-14) | 11 (7-16) |
| Mean ANC, cells/μL (range) | 4630 (960-10846) | 7968 (30-247,810) |
| Mean PLT/μL (range) | 188,125  (29000-294,000) | 181,010  (13000-540,000) |
| Median follow-up, months (range) | - | 28 (0-272) |
| First line therapy, n (%)  AML standard chemotherapy* +/- targeted agents  HMAs  BTKi  Venetoclax-based^$^  Daratumumab + RD/VD/VRD/VTD  RD/VMP/VRD/VCD  Anti-CD20 + Bendamustine  Rituximab + BAC/CHOP/COMP/CVP/FCR  TKIs  Ponatinib + blinatumomab  ATG  Eculizumab  Cyclophosphamide  Ruxolitinib  Rituximab  Pentostatin | - | 79 (71)  8 (7)  7 (6)  4 (4)  2 (2)  18 (16)  5 (5)  11 (10)  11 (10)  2 (2)  3 (3)  1 (1)  1 (1)  2 (2)  1 (1)  2 (2)  1 (1) |
| Responses to therapies  PD/SD  CR/PR  Not evaluable§ | - | 26 (23)  43 (39)  42 (38) |

*AML standard chemotherapy included: 3+7 cytarabine + daunorubicin or 2+5 +/- gemtuzumab-ozogamycin or midostaurin; cytarabine alone; cytarabine + venetoclax; and liposomal cytarabine + daunorubicin. $, venetoclax + obinutuzumab or venetoclax. §, not evaluable because: (i) ongoing therapies (N = 5); (ii) not on therapy (N = 19); not available (N = 8). Abbreviations. MGUS, Monoclonal gammopathy of uncertain significance; Hb, hemoglobin; ANC, absolute neutrophil count; PLT, platelets; AML, acute myeloid leukemia; HMAs, hypomethylating agents; BTKi, Bruton’s tyrosine kinase inhibitors, including ibrutinib and acalabrutinib; RD, lenalidomide + dexamethasone; VD, bortezomib + dexamethasone; VRD, bortezomib + lenalidomide + dexamethasone; VTD, bortezomib + thalidomide + dexamethasone; VMP, bortezomib + melphalan + prednisone; VCD, bortezomib + cyclophosphamide + dexamethasone; BAC, Bendamustine + Cytarabine; CHOP, cyclophosphamide, doxorubicin, vincristine, prednisolone; COMP, cyclophosphamide, vincristine, prednisolone, non-pegylated liposomal doxorubicin; CVP, cyclophosphamide, vincristine, prednisolone; FCR, fludarabine, cyclophosphamide, rituximab; ATG, anti-thymocyte globulin; TKIs, tyrosine kinase inhibitors; PD, progressive disease; SD, stable disease; CR, complete remission; PR, partial remission.

**Table S2. Patients’ characteristics of the cohort for neutrophil subset immunophenotyping.**

| **Characteristics** | **Healthy subjects**  **N = 21** | **Patients**  **N = 33** |
| --- | --- | --- |
| Mean age, years old (range) | 51 (25-75) | 64 (25-86) |
| Sex (M/F) | 13/8 | 20/13 |
| 2022 WHO classification, n (%)  MGUS  Myelofibrosis  CMML  BPDCN  Chronic myeloid leukemia  Chronic lymphocytic leukemia  Acute leukemias  Bone marrow failure syndromes  Autoimmune thrombocytopenia/hemolytic anemia  Myelodysplastic syndromes  Non Hodgkin lymphomas  Multiple Myeloma | - | 5 (15)  5 (15)  1 (3)  1 (3)  1 (3)  2 (6)  2 (6)  3 (9)  5 (15)  3 (9)  4 (12)  1 (3) |
| Mean Hb, g/dL (range) | 13 (7.5-16) | 12 (7-16) |
| Mean ANC, cells/μL (range) | 1997 (1070-9750) | 4351 (500-13660) |
| Mean PLT/μL (range) | 175,677  (19100-333,000) | 193,867  (14000-555,000) |
| First line therapy, n (%)  Standard chemotherapy  HMAs  TPO agonists/luspatercept  Ponatinib + blinatumomab  Ruxolitinib  Rituximab  Rituximab + venetoclax  BTKi | - | 21 (64)  5 (15)  3 (9)  6 (18)  1 (3)  2 (6)  2 (6)  1 (3)  1 (3) |

Abbreviations. Hb, hemoglobin; ANC, absolute neutrophil count; PLT, platelets; HMAs, hypomethylating agents; TPO, thrombopoietin; BTKi, Bruton’s tyrosine kinase inhibitor.

**Table S3.** **Circulating levels of reported NET-related proteins in the plasma of AA and HC [15].**

|  | **Before IST** | | | | | | **After IST** | | | |
| --- | --- | --- | --- | --- | --- | --- | --- | --- | --- | --- |
|  | **HC vs AA** | | **HC vs CR** | | **HC vs NR** | | **HC vs AA** | | **HC vs NR** | |
|  | **FC** | **t-test** | **FC** | **t-test** | **FC** | **t-test** | **FC** | **t-test** | **FC** | **t-test** |
| PRTN3 | 2.96 | <0.0001 | 2.32 | 0.0084 | 4.09 | <0.0001 | 1.51 | 0.001 | 2.07 | 0.0002 |
| LTF | 2.56 | 0.0223 |  |  | 5.51 | <0.0001 | 2.08 | 0.001 | 3.04 | 0.0019 |
| TKT | 2.50 | <0.0001 | 2.35 | 0.0007 | 2.59 | <0.0001 |  |  | 2.54 | <0.0001 |
| ELANE | 1.16 | <0.0001 |  |  | 1.16 | 0.0136 |  |  |  |  |
| MPO | 1.35 | 0.0017 | 1.11 | 0.0021 | 3.65 | <0.0001 |  |  |  |  |
| LYZ | 0.53 | 0.0047 |  |  |  |  |  |  |  |  |

**Abbreviations.** IST, immunosuppressive therapies; HC, healthy controls; AA, acquired aplastic anemia; CR, complete responders; NR, non-responders; FC, fold change; PRTN3, proteinase 3; LTF, lactoferrin; TKT, transketolase; ELANE, elastase; MPO, myeloperoxidase; LYZ, lysozyme.

**Table S4.** **Circulating levels of reported NET-related proteins in asymptomatic HLTV-1 carriers (AC) and patients with Adult T-cell leukemia/lymphoma (ATL) [17].**

| **AC vs ATL** | | | | | | |
| --- | --- | --- | --- | --- | --- | --- |
|  | **t-value** | **Degree of freedom** | **p-value** | **CI**  **(Lower Limit)** | **CI**  **(Upper Limit)** | **AUC ROC** |
| PRTN3 | -1.7708 | 49.8364 | 0.0827 | -13540.07 | 852.48 | 0.5625 |
| LTF | 0.6029 | 74.5855 | 0.5484 | -14899.25 | 27831.03 | 0.5619 |
| TKT | 2.9952 | 77.9155 | 0.0037 | 8446.91 | 41936.52 | 0.7131 |
| ELANE | -0.5336 | 51.9057 | 0.5959 | -1245.73 | 722.43 | 0.5694 |
| MPO | -3.679 | 47.9232 | 0.0006 | -26508.67 | -7772.99 | 0.7319 |
| LYZ | -8.1858 | 74.2403 | 5.65E-12 | -28300.83 | -17220.78 | 0.8925 |
| **Remission vs AC** | | | | | | |
|  | **t-value** | **Degree of freedom** | **p-value** | **CI (Lower Limit)** | **CI (Upper Limit)** |  |
| PRTN3 | -1.8576 | 5.3972 | 0.1181 | -14734.07 | 2214.28 |  |
| LTF | -1.8611 | 4.6663 | 0.1259 | -110578.53 | 18866.67 |  |
| TKT | -9.0609 | 33.3273 | 1.65E-10 | -74900.54 | -47440.49 |  |
| ELANE | -2.7049 | 6.6843 | 0.03179 | -1986.37 | -123.83 |  |
| MPO | -2.1220 | 5.4899 | 0.0823 | -18070.83 | 1488.89 |  |
| LYZ | 0.3775 | 4.3966 | 0.7233 | -18528.59 | 24602.85 |  |
| **Remission vs ATL** | | | | | | |
|  | **t-value** | **Degree of freedom** | **p-value** | **CI (Lower Limit)** | **CI (Upper Limit)** |  |
| PRTN3 | -2.8 | 16.2986 | 0.0141 | -22304.81 | -2902.58 |  |
| LTF | -1.6 | 5.04764 | 0.1773 | -103813.78 | 25033.71 |  |
| TKT | -5.2 | 34.4839 | 9.16E-06 | -50037.72 | -21919.88 |  |
| ELANE | -2.3 | 23.0599 | 0.0297 | -2491.65 | -141.85 |  |
| MPO | -4.5 | 20.2568 | 0.0002 | -37306.86 | -13556.74 |  |
| LYZ | -2.4 | 4.63439 | 0.0641 | -41189 | 1741.65 |  |

**Abbreviations.** CI, confidence interval; PRTN3, proteinase 3; LTF, lactoferrin; TKT, transketolase; ELANE, elastase; MPO, myeloperoxidase; LYZ, lysozyme.
